# Effects of weaning‐related stress on the emotional health of horses—A scoping review

**DOI:** 10.1111/evj.14412

**Published:** 2024-08-29

**Authors:** Joanne Dwyer, Amanda L. Roshier, Madeleine Campbell, Bradley Hill, Sarah L. Freeman

**Affiliations:** ^1^ School of Veterinary Medicine and Science University of Nottingham Leicestershire UK

**Keywords:** foal, horses, mare, stress, weaning

## Abstract

**Background:**

Weaning represents one of the most stressful events in the life of a horse, and may have long‐term impacts on behaviour. There are a range of approaches used to wean foals from mares, including abrupt and progressive separation methods. There is currently a lack of consensus on how stress and impact are measured.

**Objectives:**

To conduct a scoping review to identify and chart measures of weaning‐related stress on the mare and foal.

**Study design:**

Scoping review.

**Methods:**

The scoping review was conducted using the PRISMA extension for scoping reviews. Systematic searches were conducted in three scientific databases (CAB abstracts, Medline and Embase). The title, abstract and full text of retrieved studies were reviewed against inclusion and exclusion criteria. Publications that remained after full text review were analysed. Data on study design, population characteristics, weaning method and health and behavioural outcomes were extracted and charted.

**Results:**

A total of 366 publications were identified; 22 were retained for inclusion and charting. Eighteen studies reported behavioural measures, four only reported physiological measures and 12/22 studies used a combination of both. Fifteen studies analysed foal behaviour only (15/22), six analysed foals with their dams and one analysed the mare only. Duration of most studies (19/22) was a maximum of 12 months post weaning; three studies continued measurements until the foal age was 3–4 years old.

**Main limitations:**

Limited number of studies available for analysis.

**Conclusions:**

There is significant variation in the current evidence around weaning‐related impacts in the mare and foal. This includes variation in how weaning methods and management of mares and foals are described, and very limited research evaluating long‐term impacts, or the impact on the mare. This scoping review makes recommendations on how to improve the future evidence base.

## 1 INTRODUCTION

Equine behaviour is influenced by many social and environmental factors and the stress associated with weaning is considered unavoidable. In wild and semi wild herds, foals remain closely attached to their mothers until they are old enough to leave the family group to establish their own herds at 2 to 3 years old. In domestic circumstances foals are weaned from their mothers as young as 4 months old and have traditionally been isolated in a stable on their own for days or weeks until their stress symptoms subside. Although limited, the research conducted on foal weaning has identified several relevant factors that can influence best practice and therefore may improve the welfare of both mares and foals in the immediate term. There is some agreement that colts are more resilient to weaning‐related stress than fillies.^1^, ^2^ The housing and social structure that foals are managed in post weaning is an important consideration. Studies generally agree that keeping foals in familiar groups with a balance of both young and adult horses, housed in paddocks is the least stressful way for foals to be managed after separation from the mare.^3^

As yet, there have been no systematic reviews, research syntheses or scoping reviews published on the impact of weaning‐related stress on the long‐term emotional health of the horse. Therefore, decision‐making on methods for best practice remains challenging. The aim of this study was to identify and chart the current evidence on the effects of the stress experienced by the horse at the point of weaning, and to highlight areas where knowledge is lacking.

The objectives of the study were to:

- conduct a systematic search of databases using published methodology to identify current literature on weaning horses.
- review and appraise the literature on the effects of weaning horses.
- chart and summarise evidence on associations between emotional development and behavioural problems in horses following weaning.
- chart study methodology from existing publications to identify knowledge gaps and construct recommendations for future studies to improve the evidence base.

## 2 MATERIALS AND METHODS

### 2.1 Protocol and registration

A search for scoping and systematic reviews on the impact on behaviour of foal weaning was conducted in the VetSRev database^4^ on 16 October 2023 and no reviews were identified. A Preferred Reporting Items for Systemic reviews and Meta‐Analyses Extension for Scoping Reviews was used for this scoping review.^5^ The scoping review protocol was developed before data extraction and registered on Open Science Framework (https://osf.io/3gjz2). The project was reviewed and approved by the Ethics Committee, School of Veterinary Medicine and Science, University of Nottingham.

### 2.2 Search strategy

Primary literature searches were conducted on 26 March 2023 in three scientific databases: CAB Abstracts (1973–present), Medline (1996–present) and Embase (1974–present). Search terms were developed and reviewed by a team of four people, including authors and a librarian (Item S1).

### 2.3 Study selection

The publications identified in the searches were exported into Rayaan.^6^ Duplicate records were identified and deleted. Retained publications were then assessed against the inclusion criteria (Item S2). A three‐stage systematic review and exclusion process was completed independently by two researchers (JD and AR) involving (1) review of publication titles, (2) review of publication abstracts and (3) review of the full‐text for the remaining publications. At title and abstract review, ambiguous publications were retained for the next stage of review. Publications where abstracts could not be identified were retained for full‐text review. A study was excluded if English language full‐text was not available from University of Nottingham libraries or e‐libraries, from free online Open Access and legal deposit libraries, from online searches, or from direct contact with authors or journals.

### 2.4 Charting process

**FIGURE 1:**
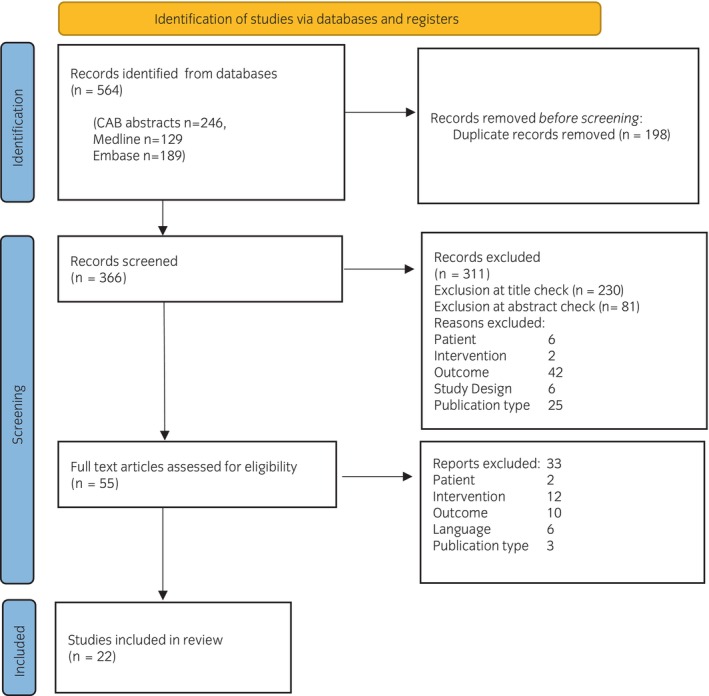
PRISMA 2020 flow diagram showing the number of publications that were identified and reviewed and reasons for exclusion from the review of the literature on the effect of weaning‐related stress on the emotional health of horses.

The remaining final full‐text publications were read and assessed independently by two researchers to ensure eligibility for inclusion. Characteristics and relevant information were extracted and presented as shown in Figure 1.

### 2.5 Data extraction

Following full text review, key information was extracted from each of the remaining 22 publications using a standardised form (Item S3) and tabulated. Study characteristics extracted and charted were: author, publication year, study design, sample size, weaning method, measurement points and duration and sample size (Table 1). Study population behavioural measures extracted and charted were: Defecation, vocalisation, locomotion, eating, lying, affiliative social interaction, agonistic social interaction, maladaptive behaviours (Table 2). This was carried out independently by one researcher.

**TABLE 1:** Study characteristics of 22 publications that were identified in a scoping review of literature on the effects of weaning‐related stress on the emotional health of horses.

Author	Year	Study design	Country	Weaning method and housing methods in the study	Measurement points and duration of measurements	Number of mare and foals
Araba and Crowell‐Davis^7^	1994	PC	US	Insufficient information on weaning method. Paddock housed mixed foal herd after weaning. Mares remained together in the paddock they occupied with the foals.	1 week old to post weaning.	15 mares and 10 foals
Christensen et al.^8^	2020	PC	Denmark	Abrupt separation Housed in groups of five colt foals after weaning.	5 months old (pre weaning), 1 year (3 months post weaning), 3 years old.	25 colts/stallions
Dubcova et al.^9^	2015	QE	Czech Republic	Abrupt separation Housed in mixed foal herds after weaning.	Up to 140 days after weaning.	56 foals
Erber et al.^10^	2012	QE	Germany	Combination of separation methods: all mares removed from group; broodmares removed from the group, but two nanny mares left behind; few mares removed from the group progressively. Foal groups all housed in large stables with straw.	The day before to 8 days after weaning.	17 foals
Falomo et al.^11^	2020	QE	Italy	Abrupt separation One group of mares' paddock housed together. The other group of mares initially individually stall housed for 2 days and then turned out into a paddock. Insufficient information on foal housing after weaning.	7 days pre weaning, day of weaning, 7 days post weaning and 30 days post weaning.	22 mares
Gorecka‐Bruzda et al.^12^	2015	QE	Poland	Abrupt separation Foals housed together in pens (5–8 foals in a pen) after weaning.	1 day pre weaning, day of weaning, 1 day post weaning.	53 weanlings
Heleski et al.^13^	2002	QE	US	Abrupt separation. One group of foals housed individually in stalls. One group of foals housed in groups in paddocks.	Day of weaning then 2 days per week 6 h per day for a total of 56 days.	12 weanlings
Henry et al.^3^	2012	QE	US and France	Abrupt separation. Foals group housed in paddocks. One group housed with the presence of two unrelated adults. One group was peers only.	2 weeks prior to weaning through 1 month after.	32 foals
Holland et al.^14^	1996	QE	US	Combination of separation methods: some abrupt, some gradual. Foals paddock housed after weaning.	Blood sampling 72 h post weaning (exp 1) and 48 h post weaning (exp 2). Behaviour measures 1 h on weaning day and the next 2 days for both experiments.	44 foals
Houpt et al.^15^	1984	QE	Unknown	Insufficient information on weaning method. Foals housed in singles and pairs in stalls after weaning.	Focal samples just before and just after weaning, then 6 h, 12 h, 24 h and 1 week post weaning. Blood samples after each observation period except the 12 h one.	22 foals
Lansade et al.^16^	2018	QE	France	Combination of separation methods: one group abrupt, one group progressive. Foals stall housed in pairs. Mares housed in groups of 4 in loose boxes with straw bedding.	Four weeks pre weaning, the day of weaning, and 3 months post weaning.	34 foals and their dams
Malinkowski et al.^17^	1990	QE	US	Insufficient information on weaning method and housing after weaning. Foals grouped by non‐weaned foals, foals weaned singularly, and foals weaned in pairs.	Just before weaning and then 4, 8, 16, 24, 32, 40, 48 h after weaning for cortisol concentrates and just before weaning and then 4 and 28 h after for lymphocytes.	20 mare and foal pairs.
McCall et al.^18^	1987	QE	US	Combination of separation methods: some abrupt, some progressive. Foals housed in wire mesh pens of 15 m x 15 m. Mares also kept in pens. Some groups were able to have fence line contact with the mares but one group was not.	5 days pre weaning, 2 days and 9 days post weaning.	21 foals
Merkies et al.^19^	2016	QE	Canada	Abrupt physical separation but one group had a reduction in nursing 4 days prior. Insufficient information on the housing of mares and foals after weaning.	Daily for 4 days after weaning.	17 mare/foal pairs
Moons et al.^1^	2005	QE	US	Abrupt separation but one group had experienced micro separations leading up to weaning. Foals individually housed in box stalls.	Separation group 2, 4, 6, 8, 10 and 12 weeks old and then the day prior to weaning, weaning day and the day after weaning for both groups.	10 mare/foal pairs
Nicol et al.^20^	2005	PC	England	Insufficient information on weaning method. Groups of up to 12 foals housed in barns post weaning.	2 weeks pre weaning, and up to 4 years post weaning.	186 foals
Nicol et al.^21^	2005	RCT	England	Abrupt separation. One group was paddock housed in peer groups, the other was barn housed in peer groups after weaning.	From age 2 weeks to age 40 weeks.	17 foals
Normando et al.^22^	2022	QE	Italy	Abrupt separation. One group weaned together stall housed in pairs, the other group 2 foals weaned weekly, again stall housed in pairs for 6 days, then paddock housed.	7 days prior, day of weaning, 7 days after and 30 days after weaning.	22 foals
Qureshi et al.^23^	2013	PC	Germany	Abrupt separation. Insufficient information on housing after weaning.	2 days before weaning, 1 day before weaning, 1 day after weaning and 14 days after weaning.	84 foals
Waters et al.^24^	2002	PC	England	Insufficient information on weaning method. Foals housed either individually or in groups post weaning.	Up to 4 years post weaning.	225 foals/young horses
Weeks et al.^25^	2000	PC	US	Progressive separation. Group housed in paddocks after weaning.	From June to September (when the last foal was weaned). Weaned at 4 months old, observed up to 6 months old.	14 mare/foal pairs
Wulf et al.^2^	2018	PC	Germany	Abrupt separation. Foals then group housed with peers initially in a barn, then in a paddock.	2 days before weaning to 7 days after.	22 foals

Abbreviations: PC, prospective cohort; QE, quasi experimental; RCT, randomised control trials.

**TABLE 2:** The behavioural outcomes that were assessed in a scoping review of the literature on the effects of weaning‐related stress on the emotional health of horses (behavioural outcomes measured in each study are shaded in green).

Paper	Defecation frequency	Vocalisation frequency	Locomotion walking, trotting, cantering	Eating time budget	Lying time budget	Affiliative social interaction grooming, suckling, touching, sniffing	Agonistic social interaction kicking, biting, distancing away	Maladaptive (stereotypical) behaviours pacing, crib biting, windsucking
Araba and Crowell‐Davis^7^								
Christensen et al.^8^								
Erber et al.^10^								
Falomo et al.^11^								
Gorecka‐Bruzda et al.^12^								
Heleski et al.^13^								
Henry et al.^3^								
Holland et al.^14^								
Houpt et al.^15^								
Lansade et al.^16^								
Merkies et al.^19^								
Moons et al.^1^								
Nicol et al.^20^								
Nicol et al.^21^								
Normando et al.^22^								
Waters et al.^24^								
Weeks et al.^25^								
Wulf et al.^2^								

### 2.6 Charting of the available evidence

There were four publications reporting on health outcomes only and these were not charted further. There were 18 publications that reported on the effects of weaning on stress‐related behaviours. These were charted according to the most commonly reported impacts on behavioural outcomes: stress‐related behaviours, aggression, physiological and social behaviours and where measured/reported data was charted to include mare behaviour as well as foal behaviour.

## 3 RESULTS

### 3.1 Study selection

The primary literature search identified 366 publications following the removal of duplicates. Of these, 230 were excluded on title review, 81 were excluded following abstract review and 33 were excluded on full‐text review. Exclusions at full stage review included 6 studies that were not available in English, 10 excluded for outcome, 3 for publication type, 12 for intervention and 2 for study population. Twenty‐two publications remained for further assessment and charting.

### 3.2 Study characteristics

The final 22 papers were from studies conducted on horses in the USA (7/22), Europe (9/22), England (3/22), Canada (1/22), various or multiple countries (1/22) or location unknown (1/22) and published between 1984 and 2022 (Table 1). Seven of the 22 studies were prospective cohort studies, 14 were quasi experimental studies and 1 was a randomised controlled trial (Table 1).

The emotional impact of weaning was frequently measured using behavioural indications of stress (18/22 studies); four studies only used physiological indicators of stress. Twelve of the studies used a combination of behavioural and physiological indicators of stress.

### 3.3 Study population characteristics

The majority of the studies involved analysis of foal behaviour only (15/22). Six analysed foals with their dams and 1/22 measured the effects of weaning on the mare only. A variety of weaning methods were used. The most commonly used method was abrupt separation (12/22). Four studies used a combination of abrupt and progressive separation methods, one used progressive separation only and 5/22 studies did not state the method of separation used (Table 1). The most common housing system reported was that foals were kept in groups or pairs immediately after weaning (13/22), in 1/22 studies, the foals were individually housed. Most studies only measured indicators of stress in the short‐term (up to a maximum of 1 year post weaning [19/22]). Only three of the 22 studies continued to measure indicators of stress into the foal's third or fourth year of age (Table 1).

### 3.4 Behavioural outcomes

Behavioural outcomes only were reported in 18 of the 22 studies. Four studies only used physiological indicators of stress. Table 2 shows the eight commonly reported behavioural outcomes that were impacted by the process of weaning: defaecation, locomotion, vocalisation, eating, lying down, affiliative social interactions, agonistic social interactions and maladaptive behaviours. The most commonly reported behaviour was vocalisation which was reported in 13/18 studies that measured behaviour. The least commonly reported behaviours were maladaptive or stereotypies (7/18). Two of the 18 studies reported data on all eight behavioural outcomes (Table 2).

## 4 DISCUSSION

This is the first scoping review that collates and describes the current literature on the effects of weaning‐related stress on the emotional health of horses. The review identifies a lack of evidence, with only 22 studies eligible for inclusion. Of those, only three measured longer‐term effects of weaning on the foals.^8^, ^20^, ^24^ Only six studies measured the effects of weaning‐related stress on mares and foals.^1^, ^7^, ^16^, ^17^, ^18^, ^25^ There were several issues identified with the existing literature, which included limited research on the impacts of weaning on mares, significant variations in study design and methodology, and a limited period of time that behaviour was assessed post weaning.

Ideally studies are designed prospectively, and subjects randomly allocated to study groups to reduce the risk of bias. Most studies in the scoping review were quasi experimental. This approach gives the flexibility to study weaning‐related behaviours in real time, but often does not account for the multifactorial nature of the process. These factors include the interactions between the weaning methods used, and the housing and social structures that mares and foals occupy pre, during and post weaning. The studies that were prospective cohort studies, mainly followed the study populations for a short period of time which limits the understanding of the long‐term effects of weaning.^2^, ^7^, ^8^, ^20^, ^23^, ^24^, ^25^ However, the short‐term impacts of weaning‐related stress are clear from the existing body of research and may be used to assess the impact that major stressful events have on the long‐term resilience of foals The maximum study population for the prospective studies was 225 foals and most were studies of small groups—14/22 studies had less than 30 foals. Study design, sample size, management style and the length of the observation period all affect study reliability and how the findings can be related to the general population of horses.

The method of weaning used can have a significant impact on the behaviours observed. The weaning of horses in the UK has traditionally been performed abruptly, meaning that mare and foal have no periods of separation from each other until the day of weaning with foals being housed individually in stables or stalls in the immediate term post weaning.^16^ There are more progressive approaches to weaning where mares and foals are given very short periods of time apart to begin with.^18^ Over several weeks and months, these periods of time are increased. This gives mares and foals time to assimilate the sense of being separated. Although progressive weaning methods have been recommended by researchers for several decades, the industry has not been quick to adopt them. There is growing agreement amongst researchers that abrupt weaning methods result in higher levels of stress‐related behaviours than more progressive and habituated approaches to separation^16^, ^18^ and group‐based housing in an outdoor paddock causes the least amount of weaning‐related stress.^13^, ^21^ The majority of the studies analysed in this evidence review did not clearly describe both the method of weaning and the post weaning housing arrangements, and these two distinct processes were often merged or crossed over in the literature. A clear description and distinction between the weaning methods is essential to enable assessment of the impacts of different methods. Further research is needed to identify the ways in which stress at weaning can be minimised to support horses through this critical time in their development. This evidence review highlights that studies related to weaning need to clearly define the methods being used, to enable robust evidence to be generated and consolidated to inform future best practice.

Research into weaning methods and how they impact the mares and foals are important. There is currently a suggestion that some maladaptive or stereotypical behaviours may be initially triggered by weaning,^20^ so assessment of behavioural traits associated with weaning is of particular interest. Eighteen studies mapped behavioural changes during weaning. The most common behaviour traits impacted by weaning were those relating to stress and anxiety, and these could be divided into eight classifications of behaviours as reported in Table 2. These behaviour classifications were reported reasonably consistently across the studies. However, most studies only focused on short term effects, and only three studies measured the effects of weaning up to 3–4 years of age.^8^, ^20^, ^24^ Measuring the long‐term effects of weaning is a complicated task because horses seldom have one owner for their whole lives. Most horses are sold as foals or young horses so this presents a significant challenge to researchers. This scoping review highlights a need for further research in this area.

The majority of studies were focused on the foal, and there were limited studies that evaluated both mare and foal. This is important both because weaning can impact the welfare of both mare and foal, and because the mare/foal interaction may be an important factor for future behaviour of the foals. One study reported that mares showing signs of dominance‐based aggression and high social rank within their herds, their foals were more likely to continue this behaviour by rank amongst their peers' post weaning.^7^ These more aggressive foals were more likely than lower ranking foals to develop maladaptive or stereotypical behaviours.^24^ However, the development of behaviour is complex and multifactorial. The impact of how foals are managed post weaning was reported to have more bearing on the manifestation of the behaviour, as the presence of unrelated adult horses appears to minimise the expression of aggression and stress‐related behaviour in foals.^3^, ^10^ The current evidence is limited by the diversity of the research, however it is clear that the impact of weaning may be affected by a number of factors including the behaviour and social ranking of the mare, the method of weaning used, the age and gender of the foal, the social grouping before and after weaning and the methods of housing before and after weaning. Future studies of weaning should be developed with these factors in mind, so that the methodologies and influencing factors are reported, and incorporated in data analysis.

The limitations of this scoping review are that the database searches were conducted in March 2023. Since that time, further studies may have become available. The search may not have identified all the available literature on the topic, the databases used were consistent with those reported as most suitable for the veterinary literature. However, the study did not include grey literature, and publications that were not available in English. The major limitation was the challenge in comparing and consolidating the current evidence due to significant variation in methodology and reporting.

A scoping review was chosen for this project due to the broad question being asked. A scoping review does not allow for the analysis of literature relating to a specific question, or the reporting of findings in a way that may be expected from a systematic review, but this was not the aim of the study. Had a systematic review been undertaken, for example, only the three studies that examined the long‐term impact of weaning would have been included, and the variations in methodology and reporting weaning methods meant these could not be compared and consolidated.

Future studies that aim to examine the long‐term effects of weaning‐related stress on horses, should clearly identify the method of weaning and post weaning management and continue to measure stress‐related behaviours into the young horses' ridden career. Other important factors that should be considered include the welfare of broodmares as they may produce several foals across their lifetime and risk being subjected to high levels of unnecessary trauma on a relatively regular basis. More research is also needed to determine whether there are differences between colts compared with fillies for the impact of weaning‐related stress, based on limited evidence documenting gender differences in two of the studies.^1^, ^2^

Studies should be undertaken prospectively, with mares and foals randomly allocated to weaning groups in which weaning and housing methods are described, and consistent, and validated methods should be used to measure behaviour, including either direct behavioural observations or validated behaviour assessment tools. Consideration should also be given to the amount of time between the point of weaning and collecting behaviour measures to ensure that the long‐term effects of weaning‐related stress are identified. Study population sizes should ensure that the minimum number of mares and foals needed for valid statistical analyses are recruited to the studies.

## 5 CONCLUSIONS

This scoping review identified and described the available literature relating to the effects of weaning‐related stress on the long‐term emotional health of horses. Only three studies measured the long‐term effects of weaning. The remaining 19 studies used such a variation of weaning and housing methods that it is extremely challenging to assess whether weaning‐related stress is unavoidable or indeed carried forward into the lifetime of the horse. Most studies were quasi experimental and varied in their methodologies, quality and means of categorising the management of horses which may have had a significant impact on the stress‐related behaviours observed in the studies.

The review has identified a lack of evidence to support the understanding of the long‐term impact of weaning on horse behaviour and emotional resilience. In addition, it has highlighted weaknesses in the existing studies because of the lack of categorisation of weaning methods, management of mare and foals, the short duration of study designs, and the lack of mare specific research. Recommendations have been made on how to improve future study designs and reporting to generate a robust evidence base which can be used by veterinarians, behaviourists, horse owners and carers, and other industry professionals when making decisions on weaning and early years management.

## AUTHOR CONTRIBUTIONS

**Joanne Dwyer:** Conceptualization; investigation; writing – original draft; methodology; validation; writing – review and editing; formal analysis; data curation. **Amanda L. Roshier:** Conceptualization; investigation; methodology; writing – review and editing; validation; formal analysis; data curation; supervision. **Madeleine Campbell:** Conceptualization; writing – review and editing; methodology; supervision; validation. **Bradley Hill:** Conceptualization; methodology; validation; writing – review and editing; supervision. **Sarah L. Freeman:** Conceptualization; investigation; methodology; validation; writing – review and editing; formal analysis; supervision; data curation.

## FUNDING INFORMATION

Not applicable.

## CONFLICT OF INTEREST STATEMENT

The authors declare no conflicts of interest.

## DATA INTEGRITY STATEMENT

Joanne Dwyer, Amanda L. Roshier and Sarah L. Freeman were responsible for the review and charting of the publications.

## ETHICAL ANIMAL RESEARCH

This study was reviewed and approved by the School of Veterinary Medicine and Science, University of Nottingham Ethics Committee.

## INFORMED CONSENT

Not applicable.

## Supporting information


**Data S1:** Search terms used in a scoping review designed to identify and chart the current evidence on the effect of weaning‐related stress on the emotional health of domestic horses.

**Data S2:** Inclusion and exclusion criteria for a scoping review of the literature to identify and chart the current evidence on the effect of weaning‐related stress on the emotional health of horses.

**Data S3:** Data extraction form for a scoping review of the literature to identify and chart the current evidence on the effect of weaning‐related stress on the emotional health of horses.

## Data Availability

The data that support the findings of this study are available from the corresponding author upon reasonable request: Open sharing exemption granted by editor for this review.
