# Supplementary material for: Effects of weaning‐related stress on the emotional health of horses—A scoping review
Source: Equine Vet J. 2024 Aug 29;57(3):546–54. doi: 10.1111/evj.14412 (PMC11982417; doi:10.1111/evj.14412)
Supplement: Supplementary file 1 — Data S1: Search terms used in a scoping review designed to identify and chart the current evidence on the effect of weaning‐related stress on the emotional health of domestic horses. [file EVJ-57-546-s001.pdf]

Equine Veterinary Journal BEVA

Supplementary Item 1:

Search terms used in a scoping review designed to identify and chart the current evidence on the effect of weaning related stress on the emotional health of domestic horses.

(horse* or pony or ponies or mare* or dam or dams or broodmare or equus or equine*).mp.
(wean* or suckl* or postwean* or ((separat* or removal) adj3 mother*)).mp.
(weanling or foal*).mp. [mp=abstract, title, original title, broad terms, heading words, identifiers, cabicodes]
(emotion* or behaviour* or behavior* or welfare* or stress*).mp.
