# Supplementary material for: Effects of weaning‐related stress on the emotional health of horses—A scoping review
Source: Equine Vet J. 2024 Aug 29;57(3):546–54. doi: 10.1111/evj.14412 (PMC11982417; doi:10.1111/evj.14412)
Supplement: Supplementary file 2 — Data S2: Inclusion and exclusion criteria for a scoping review of the literature to identify and chart the current evidence on the effect of weaning‐related stress on the emotional health of horses. [file EVJ-57-546-s003.pdf]

Equine Veterinary Journal BEVA

Supplementary item 2: Inclusion and exclusion criteria for a scoping review of the literature to identify and chart the current evidence on the effect of weaning related stress on the emotional health of horses.

CRITERIA	INCLUSION	EXCLUSION
**PATIENT**	Domesticated equines (mare and her foal)	Undomesticated equines<br>Other species<br>Other equids (Donkeys, mules, zebra, hybrids)
**EXPOSURES**	Mare and her single foal with no health issues in the foal	Papers relating to bone growth disorders or other health problems.<br>Fostered/hand reared/orphan foals.<br>Twin foals
**INTERVENTION**	Weaning and separation of mare from foal	Equines used for milk/dairy/meat farming<br>Invasive management or handling of foals likely to affect behavioural responses
**OUTCOME**	Papers including independent assessments or measures of equine behaviour and/or stress during and/or after weaning.<br>(Owner observations or survey of equine behaviour)	Studies of nursing behaviours only<br>Studies of health outcomes only (including medical conditions, parasites, immunity)<br>Student or owner opinions, perceptions or attitudes without behaviour observation or measure.<br>Studies measuring individual temperament or manageability of foals only.
**LANGUAGE**	English or papers with translation available	Translation not available
**STUDY DESIGN**	Case series, cohort, case control, cross sectional	Individual case reports, qualitative studies<br>Experimental studies of pharmaceutical or homeopathic interventions
**PUBLICATION TYPE**	Peer reviewed journals<br>Conference proceedings	Narrative, textbook chapters, reviews<br>Unable to obtain full study details (abstract or full text)<br>Non-peer reviewed journals
