# Supplementary material for: Effects of weaning‐related stress on the emotional health of horses—A scoping review
Source: Equine Vet J. 2024 Aug 29;57(3):546–54. doi: 10.1111/evj.14412 (PMC11982417; doi:10.1111/evj.14412)
Supplement: Supplementary file 3 — Data S3: Data extraction form for a scoping review of the literature to identify and chart the current evidence on the effect of weaning‐related stress on the emotional health of horses. [file EVJ-57-546-s002.pdf]

Equine Veterinary Journal BEVA

Supplementary item 3: Data extraction form for a scoping review of the literature to identify and chart the current evidence on the effect of weaning related stress on the emotional health of horses.

Title	
Authors	
Year of publication	
Source/country	
Aims	
Population	
Study design<br><br>Random allocation or retrospective	
Methods	
Intervention details	
Concept	
Outcomes	
How outcomes measured	
Key findings that relate to review question	
